# Preferences as fairness judgments: a critical review of normative frameworks of preference elicitation and development of an alternative based on constitutional economics

**DOI:** 10.1186/s12962-024-00510-x

**Published:** 2024-01-30

**Authors:** Wolf Rogowski, Jürgen John

**Affiliations:** 1 Department of Health Care Management, Institute of Public Health and Nursing Research, Health Sciences, University of Bremen, Grazer Str. 2a, 28359 Bremen, Germany; 2 Institute of Health Economics and Health Care Management (IGM), Helmholtz Zentrum München (GmbH) German Research Center for Environmental Health, Ingolstädter Landstraße 1, 85764 Neuherberg, Germany

**Keywords:** Preference elicitation, Normative health economics, Welfarism, Extra-welfarism, Health-related quality of life, Multi-criteria decision analysis

## Abstract

Preference elicitation is widely used within health economic evaluations to inform coverage decisions. However, coverage decisions involve questions of social justice and it is unclear what role empirical evidence about preferences can play here. This study reviews the prevalent normative frameworks for using population-based preference elicitation and the criticisms they face, and proposes an alternative based on constitutional economics. The frameworks reviewed include a supposedly value-neutral framework of preferences as predictors of choice, preference utilitarian frameworks that aim to maximize preference satisfaction, and substantive consequentialist frameworks that aim to maximize happiness, health, or capabilities. The proposed alternative implements the idea that indices of social value are tools for conflict resolution, rather than tools for maximization. Preference elicitation is used for validating values generated by multi-criteria decision analysis results within representative processes of stakeholder deliberation.

## Context

In the face of scarce health care budgets, decisions have to be made about which technologies should be covered by public payers. Technologies and the patient groups they are applied to differ by attributes such as health benefit, severity of disease, and costs. Thus, an important task within these decisions is to select decision relevant attributes and potentially weight them against each other. Linked to this task, an appropriate maximum price for the new technology needs to be determined. Pricing systems that provide clear signals about high priority areas can be argued to be desirable because they encourage and reward innovation in areas with significant unmet need [1].

Coverage decision makers act on behalf of the covered population, and in many western countries, the funds that are subject to the decision are collected in a compulsory manner in terms of taxes or social insurance contributions. Therefore, it has been argued that coverage decisions should be made in a way to ensure that these decisions are in accordance with the preferences of those who are covered by the public payer.

Preferences can be defined as explicit or implicit individual value judgments about superiority, inferiority or indifference regarding different decision objects. Aim of preference elicitation is to collect empirical evidence about these judgments, mathematically described in terms of a value function. Preference elicitation can relate to different objects of investigation. In health economic evaluation, these are primarily (a) alternative health care goods characterized by different levels of their relevant attributes, as is particularly the case in many discrete choice experiments (DCEs) [2], (b) alternative health states which are used to derive measures of health gains used in cost-utility or cost-effectiveness analysis [3] and (c) alternative distributions of health among different persons or person groups [4, 5]. In traditional social choice, (a) and (b) can be seen to relate to the arguments of an (a) welfarist or (b) extra-welfarist social value function and (c) to its functional form (the term “value function” rather than “welfare function” is chosen here to include both welfarist and extra-welfarist concepts of value) [6, 7, p. 141]. However, in preference elicitation used for multi-criteria decision frameworks, different ethical considerations both relating to what the relevant benefits are and how they should be distributed may be included so that the distinction between the arguments and the functional form of the social value function becomes blurred. Therefore, this study adopts a broad understanding of preferences which can relate to any of the objects in (a)-(c). Depending on the ethical theory, there are different concepts of how individual preferences could relate to the desirability of the technologies from a societal perspective. This desirability is called “social value” in the following.

At first consideration, the use of preference-related information appears reasonable not only from an economic, but also from an ethical perspective: if a person prefers apples to oranges, common principles of morality recommend that, unless there are other ethically relevant concerns, this person’s preference should be met [8, p. 4041]. However, other concerns are likely to be relevant. That a value judgment is popular does not imply that it is ethically justified [9]. Moreover, the stated value judgments can be heterogeneous, conflicting with one another and ignorant of the basic criteria of justice even if they are generally accepted [10, 11]—for example, if they involve unfair discrimination against persons with disabilities.

Also, the idea that the aim of healthcare resource allocation is to maximize some kind of consequence like quality-adjusted life years (QALYs) across the population has been challenged from a non-consequentialist perspective that holds that individual rights rather than some social aggregate should be the normative basis of decision making [12, 13]. For example, also with reference to the German Constitution, the use of cost per QALY as sole decision criterion for allocating scarce health resources is clearly rejected in the German health care system [14, 15]. As a consequence, theoretical frameworks that are developed around the idea of maximizing some kind of outcome ought to be handled with caution.

The path from information collected by preference elicitation studies to social value is thus not straightforward, and there is a need to specify whether and how preference-related information should be used to generate evidence for business and policy decisions.

Depending on the normative evaluation framework used, information about preferences can be linked to social value in different ways, and the frameworks face distinct criticisms. This study provides a brief and critical review of the three major schools of thought that are most frequently invoked in the use of preference elicitation in health economics. In Sect. 3, it develops a fourth framework oriented toward constitutional economics, where “preferences” are understood as “fairness judgments” and empirical methods are used to model consented decision principles [16]. The discussion addresses links between the frameworks, and it is argued that a combination of extra-welfarist considerations with a constitutional economic understanding of social value is best suited to the ethically reflected use of preference elicitation to generate evidence about social value for cost-effectiveness and resource allocation.

## Prevalent normative frameworks for preference elicitation

Preference elicitation has been associated with three normative frameworks: a supposedly value-neutral understanding of observable choices, welfarist models oriented to maximize aggregate preference satisfaction, and welfarist and extra-welfarist concepts that aim to specify a substantive value to be maximized.

### Ethical neutrality

The most influential school of thought for the analysis of social value in modern economics is neoclassical axiomatic utility theory. In this theory, value is represented by ranked orderings of goods assumed to be complete, continuous, and transitive [17].

Within a strictly behaviorist account, preferences can be equated with choices [18]. Then, empirically estimated value functions are not interpreted as something of normative relevance, but simply as an empirical regularity to predict choices, like a formula in physics that predicts the movement of a leaf falling from a tree. Using this interpretation, preference elicitation can be used, for example, in marketing research to predict consumer decisions about new product designs. Such an example could be the development of a new, more effective fitness tracking app based on artificial intelligence. A developer could conduct preference studies to understand which features (like goal setting, tracking metrics, social sharing, reminders, connectivity to additional devices, alongside with price) are most likely to make individuals choose the app. It is relevant for technologies which are not covered by public funding but traded on markets. Purchasing decisions can subsequently reveal whether the prediction results were accurate. Preference elicitation can also be used to assess criteria of healthcare funding decisions [see e.g. 19]. This information can be used to predict the health policy maker’s choices regarding the coverage of a new digital public health intervention like a program using a fitness tracking app. This information can be compared with true coverage decisions later.

Analyses based on the above view do not make claims about social value that go beyond market shares, or the choice behavior of individuals or groups of individuals. However, as a consequence, this view cannot provide an answer to the problem of how coverage decisions should be made on behalf of citizens or insurers, apart from informing the manufacturer’s product design and pricing decisions.

The above follows from the fact–value distinction which holds that an “ought” claim cannot be derived from an “is” claim [20, p. 211 f., 21, p. 122]. It can be argued that empirical evidence about what people choose, or are predicted to choose, has no meaning in the discussion on how scarce healthcare resources ought to be allocated because this is a fundamentally normative question [22, 23]. Without some normative bridging premise between the results of preference elicitation and the coverage decision, no guidance for the decisions can be derived, and any normative premise is no longer consistent with this first view of preferences.

### Maximizing preference satisfaction

In most preference elicitation studies, a normative bridging premise is implied. Frequently, preferences are equated with utility, which characterizes the extent to which individuals desire certain goods or, more generally, states of affairs for themselves, where it is assumed that these desires are the most relevant for assigning value to policy choices. Given that the modern concept of utility can represent any reasons for preferring in an ordering, the term “desire” is used here to denote some internal representation of the preference ranking, not necessarily a feeling or some other psychological state.

Following the premise of welfarism, the “judgment of the relative goodness of alternative states of affairs must be based exclusively on, and taken as an increasing function of, the respective collections of individual utilities“ associated with these states [24]. Following the criterion of Pareto efficiency, a health policy option adds social value if a coverage scheme featuring it is ranked higher than its alternatives by at least one individual, and ranked at least as highly by all other individuals [17, p. 2 f.].

In the case study of a fitness tracking app, preference elicitation could thus be understood as a tool to assess the desirability of a certain app design. For example, if some municipality or public health payer decides to fund a development project for a digital public health app, a work package “preference elicitation” could be included to obtain an app that is most preferred by the tax payers or the individuals covered by the health payer.

Due to problems in directly measuring and comparing utility, economists traditionally prefer to measure preference satisfaction in terms of willingness to pay (WTP) in market acquisitions. WTP is based on the idea that the good in question has been traded off with all other goods an individual could have bought from his/her limited budget. In the absence of functioning markets, methods of preference elicitation can be used to approximate choices that would have occurred from market exchange by estimating WTP for health technologies or certain attributes of health technologies [25. p. 11 ff.]. Following the Kaldor–Hicks criterion of potential Pareto optimality, it is sufficient if the winners can hypothetically compensate the losers so that the aggregate WTP for a technology can be compared with its costs to assess whether its coverage adds social value [26, 27].

As an alternative, creating social value functions has been proposed, where individual utility is aggregated on the basis of explicit value judgments. The elicitation of “ethical preferences” [28] concerning distribution (in contrast to egoistic preferences about individual utility) have been applied to determine how the valuations of different individuals should be transformed into an overall measure of social value [29, p. 148 ff.].

Preference elicitation can then be seen as a tool to maximize welfare in terms of the extent to which coverage decisions meet the preferences of those covered by the public payer. This can be taken (2.2.1) to imply the idea that preferences are undisputable tastes or (2.2.2) that “true” preferences are to be elicited, and evolve after some process of laundering or reflection.

#### Preferences as undisputable desires/tastes

Even if this view of preference elicitation contains normative premises, it is still influenced by positivism and the idea that normative considerations regarding the “appropriate” contents of preference functions are unscientific. Preferences can thus be assumed to be stable expressions of desires that are exogenous to health economics research [6]. Regarding taste, *disputandum non est* [30], so that the content of preferences is seen as something that should not be of concern for the positive task of preference elicitation [31, p. 8].

Preference elicitation in the work package mentioned above could thus, for example, be conducted in a population representative internet survey. The results of this survey could be taken as the preferences of the covered population regarding most desirable features of this app - and thus an appropriate basis for making legitimate decisions regarding this population’s scarce public resources.

However, whether preferences in terms of exogenous and stable rank orders do indeed exist, and are susceptible to measurement by means of standard discrete-choice or contingent evaluation studies, can be challenged. Thus, this view is unlikely to be a tenable normative basis for using preference elicitation to support health policy decisions.

The above obtains, first, because constructing complete, transitive, and context-independent rankings of alternatives places enormous cognitive burdens on survey respondents. This may be less problematic for a simple app. However, preference elicitation frequently involves more difficult topics. For example, if a respondent in a preference survey is faced with the question of valuing paraplegia or deafness compared with good health, he/she needs to think about how these health deficiencies would matter to him/her. These are likely to matter in different ways—for example as a result of their effect on his/her mental state, such as anxiety or depression, their direct effects on overall well-being, effect on the projects and activities he/she can pursue successfully, and the benefits and burdens he/she imposes on others [31, p. 17 f. and p. 120 ff.].

Second, individuals can err: In the example of the app, individuals can overestimate the app’s effectiveness, or underestimate potential negative impacts of a publicly funded fitness tracker like obsessive behavior, anorexia or burnout. In the example of valuing health states, the individual may rely on false information regarding these effects, or the values the respondent relies on may not cohere with one another or with the individual’s overall values. In imagining a life with the disability, the individual may be mistaken as to how much he/she may be able to adapt to the situation. People can have mistaken beliefs about how good things are for them and, because of these mistakes, prefer things that are in fact to their detriment.

Moreover, people might feel that they have two very different “selves,” and therefore may hold conflicting preferences, such as an immobile person’s preference for continuing sedentary behavior, on the one hand, and his/her preference for starting fitness exercises, on the other. Preferences may thus be rationally indefensible and mistaken, and individuals who have to make actual choices, such as whether to undergo an elective surgery, typically seek advice from related parties and experts, and modify their preferences in response to this advice [31, p. 123].

Third, results from cognitive psychology and behavioral economics also suggest that preferences are not given, relatively fixed traits that can be empirically elicited by a neutral, scientific observer from the outside. Instead, the evaluative attitudes that drive choices are typically fluid and shifting, and the rankings they imply strongly and systematically depend on the context and process of deliberation. For example, prospect theory describes how individuals typically anchor their evaluation of alternatives in reference to the status quo: Losses are weighted more heavily than gains, with diminishing importance of both gains and losses [32]. Moreover, a large amount of empirical literature provides evidence about preference reversals that occur alongside choice tasks, suggesting that individuals do not evaluate options according to a pre-defined preference structure, but that this structure is developed during the choice process [31, p. 108 ff., 33]. Empirical analyses in psychology have reported a number of additional cognitive errors and biases that are also relevant for valuing the benefits of a fitness tracker. For example, it is cognitively challenging for individuals to deal with small probabilities or assign monetary values to small risk reductions [34–36], even if these are frequent outcomes of medical interventions that may need evaluation.

Fourth, preferences may result from manipulation or distorting psychological processes [37, 38]. For example, deprived populations may adapt to their situation and cease to express preferences for social states they could legitimately claim from a viewpoint of justice [39]—as in the case of socio-economically deprived population groups who have adapted to health problems, like neck or tooth pain, and do not expect them treated for them on par with people from more affluent parts of society.

#### Preferences as rationally reflected rankings

To address some of the above concerns, it has been proposed that, rather than with the satisfaction of actual preferences, social value should be equated with some type of “laundered” preferences [38]. Harsanyi has claimed that the satisfaction of preferences should relate to a situation in which a person is informed by the relevant empirical evidence; where he/she has considered all relevant aspects with due diligence, and where he/she is in an appropriate mental state to make the decision [40, p. 55, 41, p. 102].

However, this view involves difficult value judgments about the preferences that are “laundered” and empirical challenges about how such counter-factual constructs should be measured. One argument that motivates the concept of decision utility (i.e., utility is expressed in preference rankings) rather than hedonistic utility (i.e., utility is a state of the mind, see Sect. 2.3.2) is that the latter is not susceptible to empirical analysis. The same may be the case for the “rationality” of preferences [42, p. 226 f.]. It is difficult to determine how the preference elicitation project for a fitness tracker would have to be conducted to ensure that it captures “laundered” preferences– and, very likely, funders would hesitate to commission such a project because it would involve much more money and respondent time than standard preference elicitation projects.

Further, for both the concepts of preferences as undisputable desires and preferences as reflected rankings, the view that policy choices should maximize aggregated preference satisfaction can be associated with preference utilitarianism [41, p. 101 f.], a view that is susceptible to criticism. In particular, allocating social resources to maximize aggregated preference satisfaction without actual compensation can lead to morally counter-intuitive results, e.g., in the case of expensive tastes or anti-social preferences.

Furthermore, even if neoclassical welfarism is the dominant normative school of thought in academic economics, the use of WTP for health economics evaluation has been criticized for many reasons. One important objection concerns distribution: WTP is a biased measure of preference satisfaction for normative uses that involves interpersonal comparisons across people with different abilities to pay [6]. In the example of the fitness tracker, it is well established that those populations in society that are of higher risk of obesity and that might thus be considered of higher need for such a publicly funded app have lower average incomes and thus lower ability and willingness to pay. In general, methods have been developed to account for distributive concerns, such as weights to correct WTP estimates for deviation from the average income [43]. However, such corrections are rarely made in practice, and involve additional value judgments that go beyond purely positive preference elicitation [17]. It is also unclear whether such weights can be considered utility-related information [6].

The use of aggregated social value functions based on ethical preferences has also been criticized. It is unclear what ethical preferences are, as opposed to “normal” preferences—the benefit of others can be part of an individual’s preference rankings, for example in the case of a parent who also cares for his/her child. Moreover, the idea that states of affairs can be judged according to their goodness from a neutral or a societal perspective can be shown to suffer from a fundamental category error: It applies the individual conception of rational choices to questions of justice. Judging a social state as “better” always involves a definition of whom the social state is better for, and “society” is not a morally relevant entity for one to whom this case would apply [29, p. 155 ff.]. In general, as Rawls has argued, desires and wants are not by themselves reasons in matters of justice: “The fact that we have a compelling desire […] does not argue for the propriety of its satisfaction any more than the strength of a conviction argues for its truth” [44, p. 190].

### Maximizing a substantive value

Klonschinski assigns the category error described in the previous section to the fact that, despite all efforts to develop an axiomatic concept of utility, modern welfare economics is still deeply influenced by the earlier concept whereby the utility to be maximized is actually some substantive good [13]. Different substantive suggestions have been made to characterize the axiology, i.e., the ethically justified “good,” which is to be described by preference elicitation and maximized in decisions on scarce (healthcare) resources. These axiologies include health, happiness, and capabilities.

#### Preferences as evaluations of health

Typically, (economic) evaluations of new medical interventions, like drugs to combat Alzheimer’s disease, assess health benefits directly. This can be associated with the notion that the task of health policy makers is to improve health rather than utility derived from healthcare interventions. Hence, an extra-welfarist evaluation framework is required that incorporates other measures of benefit beyond individual utility [6, 45].

The extra-welfarist framework of the healthcare decision maker assumes that coverage decisions are to be made to produce as great a set of health outcomes as possible from a limited healthcare budget. To account for quality of life, episodes of disease need to be valued to produce some weighted index of health gain, which can then be aggregated to determine total social value generated by the health technology under consideration. A frequently used measure of health gain is the quality-adjusted life year (QALY). An intervention can be evaluated by comparing its cost per QALY with a threshold value that estimates the costs per QALY forgone elsewhere in the system [46].

In the case study above, preference elicitation would thus not be conducted for the fitness tracking app directly. Instead, preferences would be used to aggregate different dimensions of health outcome to one health index. This index could then be used by a healthcare decision maker for cost-utility analyses that compare the app’s cost per health gain to the cost per health gain of other interventions.

Even if QALYs can also be assigned a welfarist interpretation, according to which they can be regarded as utilities [47], this interpretation requires stringent assumptions that are unlikely to hold [48]. Therefore, in their most explicit use by the UK National Institute of Health and Care Excellence (NICE), QALYs are seen as an extra-welfarist measure of health gain [49]. Preference elicitation can be used here to provide an estimate of how severely a condition affects health and, thus, what quality weight is most appropriate to assign to it.

A similar view was adopted by the World Health Organization when revising their estimates of disability-adjusted life years conducted during the 2010 revision of the Global Burden of Disease study. In large-scale preference elicitation studies, individuals were presented with two hypothetical people with different health states, and asked which one they regarded as healthier [50].

Given that simple maximization of health outcomes may conflict with ethical principles like concern for the worst off, distributive concerns can be added by attaching so-called equity weights to the relevant outcomes. For example, health gains that accrue to patients in particularly severe conditions are typically assumed to be more valuable, and health gains in such patient subgroups can be adjusted accordingly [6, 51]. Preference elicitation can also be used for these tasks, and there is a growing body of literature regarding distributive preferences that can be used to optimize distributive policies [5].

However, a number of instruments for the measurement and preference-based valuation of health are currently available—for example, the EuroQoL, 5 Dimensions (EQ-5D), the Health Utilities Index Mark 2 (HUI-2), and the Short Form, 6 Dimensions (SF-6D) questionnaires [3]. The evidence suggests that these instruments partly measure different constructs, and are correlated with different measures of individual well-being to a varying extents. In addition, some of the health measures may lead to a systematically lower evaluation of certain classes of health services, in particular mental health services [52]. Taking the case study above, a fitness tracking app could motivate individuals to exercise, or it could additionally enable individuals to build a community of exercising friends. Potential additional benefits of the second app on social aspects of health are likely not to be captured by measures like the EQ-5D that focuses on individuals. This illustrates that until now, no unanimous answers are available to the questions of what health is and how it can be measured.

More fundamentally, it can be argued that “health” does not exist as an entity that can measured and summed up in a naturalistic manner. Even if diseases typically involve stages of varying severity, “disease” always involves disease entities that are, by definition, unequivocal, distinct, and mutually exclusive [53]. Quantifying health and disease for economic evaluation is an evaluative exercise and thus requires a normative theory, for which aspects of disease are relevant to the evaluation.

#### Preferences as evidence of happiness

The traditional normative view is that rather than preference satisfaction, social value is best understood as the aggregate of subjective well-being. One account of what well-being consists of is provided by Jeremy Bentham’s hedonic utilitarianism, which formed the basis of neoclassical economics in the 19th century. This can be seen, e.g., in Edgeworth’s definition of happiness as the integral of duration of enjoyment times the degree thereof, which should be multiplied by the number of individuals experiencing the enjoyment [54, p. 57]. Hedonistic utility is currently experiencing a renaissance in the scientific literature as a measure of subjective well-being [55].

According to this view, happiness is usually understood in terms of contentment or “life satisfaction.” People try to make choices that make them as happy as possible [56]. This view addresses the problems of inconsistent choices, failures to learn from experiences, and other potential limitations of choices as mere rank orders. Such instruments as life satisfaction questionnaires, evaluated time use, or the “U-index,” which measures the proportion of time an individual spends in an unpleasant state, have been developed to achieve this goal [57]. Moreover, measures of subjective well-being can be extended beyond hedonic pleasures and pains toward such experiences as engagement, purpose, or meaning [58].

The idea would thus not be that the decision makers assess cost-effectiveness in terms of the fitness tracking app’s cost per health gain with that of other interventions. Instead, the comparison would be how cost-effectively it makes the covered population better off in terms of happiness. Here, the app case study which additionally empowers an individual to build an exercising community is likely to perform better.

However, even if conceptualizing the aim of preference elicitation in a substantive manner, such as by maximizing subjective well-being, provides some objective basis against which decision biases can be compared, these substantive measures are also subject to measurement challenges. For example, currently available measures of subjective well-being struggle with memory biases, systematically differing interpretations of response scales by individuals in different health states, or focusing-type bias [59].

Mental state theories of social value can also be criticized more fundamentally: Suppose an individual is happy because he/she mistakenly believes that his/her life projects are going well, maybe just because the person is suffering from Alzheimer’s dementia. Suppose there is an intervention that can cure the person instantly for free; as a consequence, the person’s life projects start faring better but he/she is less happy owing to a more realistic view of the world. From a medical perspective, this intervention would have a high value, even if happiness may have declined [31, p. 79].

Finally, it should be stressed that the methods of preference elicitation currently used by health economists to value health states do not directly include the notion of happiness. Valuing health states in terms of happiness occurs only indirectly if preference elicitation is conducted to assess experience utility in patients [55]. Therefore, it remains an open question whether a health state preferred by a respondent to another actually is associated, or assumed to be associated, with greater happiness for him/her.

#### Preferences as evaluations of capabilities

Evaluative frameworks addressing the normative significance of health frequently incorporate the idea that health and medical needs have a normative significance because they bear on the opportunities individuals have to pursue their life plans. Health and medical needs should be considered in decisions about funding new health technologies because government-funded healthcare payers ought to promote equal opportunities for their citizens [60].

Hausman contrasts the concepts of the “private value” and “public value” of health [61], and claims that the value of health should be assessed from the perspective of a liberal facilitator state [62, p. 161]. From this perspective, the question is not how much a health state contributes to an individual’s preference satisfaction or well-being, but rather to what extent a health state constrains the individual’s possibilities of living well and pursuing valuable objectives: “An individual asks, ‘What range *of those things that matter to me* is available to me?’ while from the perspective of the liberal state, the question is, ‘What range of worthwhile activities is available to members of the population?’” [62, p. 160].

One concept of the normative framework that incorporates the idea of equal opportunity from a public perspective, and has garnered growing interest as a theoretical basis for extra-welfarist health economic evaluation, is Amartya Sen’s capabilities approach [63]. Viewing health state weights as descriptions of capabilities assumes that disease is normatively relevant to the extent that it reduces a person’s freedom to pursue valuable acts or reach valuable states of being [64]. Moreover, health states need to be valued in terms of their impact on capability, which can be done by preference elicitation.

Despite calls to integrate the capabilities approach into health economic evaluation, proposals for its empirical implementation are still scarce. One approach is provided by Bleichrodt, who proposes the use of preference elicitation to value menus of potential functionings that individuals can choose from in the future, rather than valuing the functionings directly [63].

Following Hausman’s concept of public value, a welfare state also has duties of care and compassion [62, p. 163 ff.] and ought to evaluate the suffering a health state imposes to an individual [62, p. 170]. Therefore, he proposes a classification of health states based on the two dimensions of, first, subjective feelings associated with health states and, second, the extent to which the health states limit the range of important activities the individuals can pursue. These classes should then be valued according to how seriously they limit the range of objectives and lives of individuals [61].

However, there is still a lack of case studies testing these approaches. It is important to note that following Hausman, the mean values of representative preference elicitation studies have little meaning. Instead, it is better to conduct ranking in deliberative groups and in addition to the valuation. The reasons for these valuations should also be included in the analysis [63, p. 151 f.].

## Preferences as fairness judgments

It is likely that the value (well-being, health, or capability) generated by a health technology remains dependent on the measurement instrument used. Perhaps no convincing argument can be made to determine a single correct instrument. Despite all substantive considerations, there is thus a different category that is important to the normative relevance of the results of evaluation: The relevant stakeholders have to *agree* on some method for measuring and valuing the relevant aspects of health technologies.

For example, if a public payer’s decision about funding a fitness tracking app was taken to court in countries like Germany, it would hardly be assessed whether the decision was made to maximize preference satisfaction, health, happiness or capability. Instead, it would be assessed whether the principles and procedures of legitimate decision making on public resources were met.

The idea that obtaining social value is a matter of agreement rather than aggregation can be expanded to a fourth normative framework that has implications for preference elicitation.

### Ethical orientation

Ethically, the idea that decisions about healthcare resource allocation should be oriented at any measure of either aggregated preference satisfaction or aggregated relevant outcomes (happiness, health, or capabilities) has been criticized by the notion that just allocation of healthcare resources is a matter of fairness, which is a deontological concept and incompatible with consequentialist frameworks of outcome maximization—not the unit of value per se and its allocation, but respecting the dignity of patients is relevant for fair decision making. This requires health coverage decision makers to view the insures as ends in themselves rather than simply as a means to the end of the production of social value [12, 13, 29]. This does not mean that outcomes are irrelevant: Certainly, it is an ethically relevant aim of healthcare to produce health outcomes. However, fair resource allocation requires something different from aggregating (health) benefits across individuals. Therefore, this criticism has also been referred to as “nonaggregationism” [12].

The differences between these viewpoints are illustrated by the fact that concepts of utility theory, like utilities, benefits, and values, that pertain to healthcare coverage decisions are not easily translatable into legal categories, like claims, rights, duties, and contracts [12, p. 84]. This is visible, for example, in the fact that, even if most would agree that a disability is a detrimental health outcome, and that preventing it is typically considered a benefit, many would agree that assigning a lower value to preventing the death of a disabled individual than to preventing that of a healthy one is not acceptable. Consequentialist theories hold that deontic categories like “claims” or “rights” are posterior to, and to be explained by, the outcomes they entail. However, if there are fairness considerations that determine whether the diminished quality of life with a disability should be considered or ignored, depending on the context of the decision, then the justification follows the opposite direction [12, p. 224].

These concerns may also be reflected in inconsistencies reported in empirical studies on prioritization preferences. For example, the orders of preference elicited from person trade-off questions failed to meet the criterion of multiplicative transitivity [65]^1^. This might be because in fact, the respondents did not engage in compensatory decision making about different outcomes to be described by the methods of preference elicitation; instead, they might have used other categories of fairness with a notion of “equal chances” or “equal entitlement to treatment” [13].

A deontological ethical theory of fair healthcare resource allocation remains to be developed and justified, and there are multiple considerations from a long history of ethical and legal thought that it could be based on. Many ethical theories that have been developed to support deontological considerations are versions of contract ethics. They assume that moral commitments are best understood as resulting from hypothetical contracts among individuals who pursue “the fundamental agent-relative idea of living with others on terms of mutual respect” [Darwall 2003, cited in: 66, p. 1278]. Contract ethical theories provide different strategies to determine the principles that govern the terms of such mutually respectful interactions. One well-known example is Rawls’ idea of free and equal citizens who select principles from behind a veil of ignorance [66, p. 1278]. Rawls’ theory of justice also forms the starting point of Daniels’ ethical theory, which provides a set of criteria of accountability for reasonableness in healthcare resource allocation, i.e., criteria that aim to ensuring that decisions are made in a way that shows respect to all stakeholders affected by them [60, p. 116].

### Economic theory

Contract ethical theories can also be associated with a counterpart in economic theory. Rather than an economic framework to measure and aggregate individual preferences or some substantive axiology, social value can be analyzed in a constitutional economic framework based on the notion of mutual agreement [16].

Constitutional economics is strongly influenced by the work of Buchanan, who recommends placing the human “propensity to truck, barter, and exchange one thing for another” [Adam Smith, cited in: 67] at the center of economic analysis. Rather than a technical view of choice and optimal resource allocation, which is a matter of mathematical optimization once the social welfare function has been defined, this view of economics focuses on the analysis of productive *social* interaction characterized by behavior like “exchange,” “trade,” or “agreement” [67]. The aim of economics is to help understand productive interactions and support the design of institutions that help overcome failures of interaction [68, p. 281 f., 69].

An important element in constitutional economic analysis is to distinguish between choices within rules and choices of rules. Rules constrain individual freedom. Therefore, it needs to be explained why individuals should voluntarily choose or consent to rules at all. Buchanan’s answer is that even if rules reduce the range of actions available to individuals, at the same time, they can increase the scope of possible cooperation because they reduce the costs of preparing, negotiating, and implementing such cooperation. The best illustration of this problem is the game-theoretic situation of the prisoner’s dilemma, in which players can attain a Pareto improvement by consenting to some enforceable rule that prohibits defection [69, p. 24, 70].

In line with Kant’s concerns about the use of individual ends as a basis for legitimate legal claims, this framework does not attempt to aggregate individual utility into some overall measure. Efficiency is not considered measurable in terms of some aggregated mean benefit. By contrast, in this view, mean values of preferences can be considered meaningless because individual ends are incommensurable, and no “mean individual” exists whose preferences are met. Mean values derived from preference elicitation studies may even specify a preference function held not by a single individual within the surveyed population [71, p.51]. Assessing and averaging over choices within rules can be seen as an inappropriate object of investigation; instead, questions of coverage determination are related to the choice of rules [72]. Efficiency here simply comprises mutual and voluntary consent to an interaction, and the social or private contract on which it is based [67, 69, p. 183 ff.].

Recently, this idea has been developed to show how using cost-effectiveness in decisions about funding new health technologies like the fitness tracker app can be interpreted as an institution to overcome societal conflicts within a social statutory health insurance contract [73]. Both disadvantaged patients and affluent healthy individuals can be argued to share interests in a societal contract to provide healthcare technologies based on progressive funding. Using cost-effectiveness analysis in health care coverage decisions can be interpreted as a means of conflict resolution if the analysis is based on consented criteria to ensure the contract’s financial sustainability and to avoid implicit rationing or unaffordable rates of contribution. In this view, the economic analysis does not focus on the question of how to aggregate individual preferences, or how to specify a social value function based on some consequentialist ethical considerations. Instead, it aims at searching for a rule that can find consent by all covered under the rule [73].

There are a number of reasons for why members of a public healthcare system could consent to a rule of basing coverage decisions on health economic assessment using a specified index of social value (rather than leaving limit-setting to ad-hoc decisions by potentially different decision-making bodies). For example, compared with a situation of resource scarcity but no criteria for economic decisions, systematic economic assessment of health may increase each individual’s expectation of receiving high-priority care in case it is needed. This is because implicit rationing can easily lead to a situation where even high-priority care is withheld in an unpredictable and translucent manner. Furthermore, in case of interventions that prevent conditions that are a similar but a statistical threat to all health insurance members (e.g., the fitness tracker app for preventing common diseases like high blood pressure or diabetes), even a purely health-based outcome that maximizes the evaluative framework is likely to find a consensus. This is because regardless of the number of aggregated health outcomes, it maximizes the expected health benefit to each individual. It needs to be remembered that such a consensus is likely to be context dependent. The more the maximization rule disadvantages certain groups in society, the less likely a consensus is—e.g., in the case of the unreflective application of this idea to treatments of individuals suffering from end-stage disease who cannot be expected to benefit from long-term survival gains, or for disabled individuals whose health outcomes would be subjected to a systematic markdown [see also: 12, p. 234].

### Concept of preference

One central aspect in this guidance is how different dimensions of health benefit (e.g., improvements in mobility compared with improvements in mental health) are to be accounted for, as well as how context-specific aspects are to be factored in, like the patient’s degree of suffering at the point of care or the severity of the disease from a lifetime perspective. If the social contract does not provide a benchmark to answer these questions, the answers are based on qualitative reasoning and intuitions of the decision committees alone. In this case, it is unlikely that the decisions are made in a consistent manner, in particular when it comes to prices for medical innovations. Moreover, given the numerous deficiencies in individual cognition and potential dysfunctionalities of group decision processes, there is a risk that these decisions are not made in a reasonable manner, but instead on case-by-case political contingencies. Preference elicitation can be seen as a quantitative tool to address these barriers to fair decision principles that can find consensus.

In the standard welfarist case, preferences represent self-interested desires. However, it is possible for a rational individual to make a statement, such as “for me personally, policy A would be better; but because of fairness concerns, I’d prefer policy B to be funded.” For example, an individual may wish to prioritize healthcare funding for children with severe hereditary diseases over providing free access to preventive services, such as yoga courses, even if, as an adult without children, the individual would personally benefit more from the yoga course. Preferences thus do not necessarily imply self-interest [74, p. 20 ff.]. Further, if a respondent rates technology A over technology B, this does not imply that he/she estimates and compares units of outcome, as would be assumed in frameworks maximizing substantive value. Instead, a preference can also be a *fairness judgment*: He/she may simply feel that preferring A is the fairest way to decide in this situation from an impartial viewpoint, which is likely to be similar to how he/she would wish that the decision about his/her case should be made. Given that at least in Germany, social law and rules for determining the coverage of new technologies by public payers are strongly based on individual claims and fairness concerns [15], this view may be a more suitable economic theory of Hausman’s concept of “public value” [62, p. 161] than consequentialist theories of value.

As severity of disease, medical benefit, and costs are widely accepted criteria in the ethical literature on healthcare resource allocation [75], such criteria as an intervention’s incremental QALY gain, incremental costs, and severity suggest themselves for inclusion in a decision algorithm. Moreover, value functions that rank decision objects (use of health technologies) in a complete, reflexive, and transitive order are consistent with such a view, given that fairness requires consistent decisions across all individuals.

### Aim of preference elicitation

According to this view, the use of methods for preference elicitation can be seen as a means to further specify the decision rules in a way that can find consensus. This does not only relate to the choice of criteria, and of how they should be measured, but also to the weights that different measures of health needs, health benefits, and costs should have.

Rather than anonymous surveys, this is likely to require deliberative groups so that this information is based not only on the individuals’ relative evaluation of the different criteria, but additionally on considerations by representatives of different relevant stakeholders (e.g., elected policy makers, payers, the public, physicians, and patients) and experts (e.g., medicine, ethics, economics, and law), the criteria and weights for which are best suited to find consensus.

Population-based surveys may provide valuable external validation to such deliberation processes because they can provide evidence about preference weights typically held within the population as well as evidence about the heterogeneity of preferences. Within small deliberative groups, various social dynamics may unfold—for example, the results may largely be determined by the idiosyncratic views of single group members with particular social status or communication skills. Therefore, there is a need to externally validate the group results. However, from this constitutional economic view, evidence generated from population-based preference elicitation is not the “true” valuation, but rather provides a benchmark against which group deliberation needs be justified in case the result of the group is not consistent with that of the population.

### Concept of social value

Consistently with Buchanan’s view of what economists should do [67], social value in this view involves *agreement rather than aggregation* across individuals: not statistical mean values derived from population-based surveys per se nor the maximum of some anonymous outcome, but agreement by those covered under a scheme, or a group of stakeholders that can serve as a suitable representative relevant to the decision framework.

A single coverage decision adds social value if it is consistent with the principles of the decision framework consented to. As far as it has been possible to develop one quantitative and generalizable framework, it may be possible to quantify this social value, e.g., in a monetary figure. However, just like ordinal utility information, this figure has no substantive value, and using the figure resulting from the framework (e.g., a priority score to be compared with a threshold of minimum priority for funding) is not for the purpose of maximizing something (e.g., some aggregated priority “outcome”), but for supporting equitable decision making.

Given that the framework is likely to involve exemptions and context specific modifications [76], it may be restricted to partial rankings in specific areas of application. Furthermore, such a fairness-based framework may contain elements that are at odds with approaches to maximizing aggregated utility or extra-welfarist outcomes, e.g., due to the inclusion of transfer payments or exclusion of unrelated future costs [77]. Finally, to complement unavoidable blind spots and deficiencies of quantitative frameworks of evaluation, it may include formal procedures for deliberation and conflict resolution as complements to the substantive principles. Value is determined by the fact that introducing the complex arrangement of decision principles, and complementary rules and procedures can help find consensus and, thus, is Pareto superior to the status quo—not in terms of some quantitative measure per se. Table 1 provides an overview of the frameworks, summarizing the normative framework, its ethical orientation, economic theory, concept of preference, aim of preference elicitation, concept of “social value,” and challenges to it.

**Table 1:** Overview of frameworks for preference elicitation

Normative framework	Ethical neutrality	Maximizing preference satisfaction		Maximizing substantive value			Meeting principles consented to
**Ethical orientation**	Ethically neutral / behaviorist position	Preference utilitarianism		Health-related consequentialism	Utilitarianism	Capability- oriented consequentialism	Contract ethics / Nonaggregationism
**Economic theory**	Neoclassical axiomatic utility theory			Health-related extra-welfarism	Hedonistic utility theory	Capability-related extra-welfarism	Constitutional economics
**Concept of preference**	Ethically neutral predictor of choice	Undisputable desire/taste	“Laundered”/ “rational” desire	Evaluation of health state	Evidence of happiness	Evidence of capabilities	Fairness judgment regarding priorities
**Aim of preference elicitation**	No elicitation of “preferences” but prediction of choice	Measure strength of preference satisfaction (potentially including ethical preferences)		Parameterize health-related quality-of life measure	Provide evidence of happiness	Parameterize capability measure	Consistency checks of multi-attributive concepts of value
**Concept of social value**	N.A. (predict market share)	Aggregated preference satisfaction		Aggregated sum of (weighted) health outcomes	Aggregated sum of happiness	Aggregated sum of achieved capabilities	Consistent decisions according to rules consented to
**Challenge (example)**	No normative guidance for social decision	Doubtful that gut feeling leads to consistent, rational rank orders	Difficult to determine “laundered” and to justify “strength of preference”	Ambiguity of health measures	(Neoclassical) concerns against hedonistic utilitarianism	Concerns against consequentialism, lack of operationalization	Lack of substantive evaluation criteria, difficult to establish consent

## Discussion

Methods for preference elicitation are widely used in cost-effectiveness analysis for resource allocation decisions. However, the normative basis of their use is ambiguous. Prevalent concepts of using preference elicitation to predict observable choices, assess and aggregate self-interested rank orders, or elicit information about specific aspects of ethically justified axiologies all face limitations. As a theoretical alternative, a constitutional economic view of preference elicitation as fairness judgments was proposed here that interprets economic frameworks to measure indices of social value as means of conflict resolution.

In this framework, methods of preference elicitation are used to develop evaluation frameworks that can find consensus among stakeholders of a given decision. Just as substantive and procedural approaches to ethics are frequently seen as complementary in ethical debates, the constitutional economic approach, oriented at procedures and other substantially defined evaluation frameworks, can serve as a complement.

Given the observations concerning healthcare coverage decisions that lead to the development of extra-welfarism, it is unlikely that a framework based on WTP would find consensus. Instead, it is likely to involve criteria associated with health needs, medical benefits, and costs, which are criteria frequently found in frameworks for prioritizing health services [75], such that the combination of an extra-welfarist evaluation framework with constitutional economic considerations about process and consent may be most appropriate. The discussion of extra-welfarist evaluation frameworks has illustrated that a number of issues remain to be resolved in them, such as the justified choice of health measures, acceptable substantive adjustments for severity concerns, and deontological concerns. The combination proposed here may provide an approach to address these concerns.

Even if health resource allocation using a new digital public health intervention has been used as case study here, this framework can be applied to various indices of social value—from analyses dedicated to only comparing institutions, regions, or countries in terms of specific characteristics (e.g., health status, economic development, and environmental sustainability) to the economic evaluation of policies beyond the traditional cost–benefit analysis.

### Implications for preference elicitation

Further research is necessary to explore the implementation of the proposed framework to preference elicitation. By using the valuation of health-related quality of life for health economics evaluation as an example, this approach can be implemented in a two-stage process of preference elicitation. In the first step, techniques of multi-criteria decision analysis (MCDA) can be applied within a deliberative process of stakeholder discourse to determine the criteria and weights for healthcare coverage decisions. Recently, such methods of MCDA as PAPRIKA (Potentially All Pairwise RanKings of all possible Alternatives) have been developed that can be used both in a deliberative group setting and population-based preference elicitation studies [78]. This tool has been validated and used for generating value sets for the generic health measure EuroQol 5D 5 L for the population of New Zealand [79]. It can also be used to generate consensus about the weighting of health attributes as well as further relevant aspects in the context of using health measures in decision making (e.g., distributive weights). The results of the consensus conference can then be used as a basis to design a population-based survey similar to the study in New Zealand, in which methods of statistical inference were used to assess whether the group results were consistent with values drawn from the population.

How exactly this is implemented statistically remains to be developed. Given that the aim of preference elicitation is to externally validate the results of group deliberation, quantile regression (in particular, regression to the median) rather than regression to the mean may be required because it seeks to represent voting on a preference function rather than estimate its mean value. In addition to agreeing on weights for the multi-criteria index of social value, the deliberative groups could be asked to specify bounds of equivalence for these weights. Statistical tests for equivalence could then be used to assess whether the weights correspond with those of a pre-specified proportion of the population (e.g. a supermajority of 2/3).

Population-based preference elicitation can thus be used in a welfarist economic framework, not to assess and aggregate individual preferences, but to confirm whether or not consensus is likely to be shared by the population on behalf of which it has been made. For example, the use of population-based preference elicitation and assessment of the mean results might lead to a situation in which small minorities do not appear. A precedent stakeholder consensus process may be suited to identify and address such issues in advance. Population-based results may be biased, and are most likely not based on reasonable reflection. By means of the precedent advocatory discourse, the proposed weights have undergone challenge and dispute resolution, are informed by the opinions of the relevant experts, and incorporate the value judgments of the relevant stakeholder groups. This validation is bidirectional because the population-based study also reveals whether the stakeholder discourse met its aim of developing a framework that can meet consensus.

This approach has the advantage that it may be closer to policy processes where the elected policy makers, whose task is to act as representatives of the population, can take part in these consensus groups, and not only develop an understanding of the methods of evaluation, their implications, and the stakeholders’ views on them, but also facilitate rule-setting, which is based on political legitimacy.

### Limitations

The application of constitutional economics to the healthcare system remains novel. It is limited, for example, by the fact that its model of interaction focuses on the game-theoretic situation of the prisoner’s dilemma, whereas in healthcare, complex institutional settings and conflicting interests have evolved that can only be captured to a limited extent by such a simple model {Rogowski, 2022 #20,722}. Nevertheless, this approach has been considered appropriate because the view of economics as exchange is better suited to incorporating the political nature of health policy decisions than frameworks that are oriented at aggregating atomistic individual choices, or at establishing the objective function of an autonomous social decision maker and, thus, ignoring the necessity of political interaction.

This study proposed an approach associated with ethical contract theory as a deontological response to fairness concerns about consequentialist extra-welfarist evaluation frameworks: Not some axiology that is maximized, but respect for people, realized in voluntary consent, forms the normative basis for the use of preference elicitation. It can also be argued that this notion of fairness involves fairness concerns: Socially weak groups may be forced to consent to a contract even if it leaves them in misery because, without it, they would be left even more miserable. One frequently discussed example is a slave who “voluntarily” agrees to work for his/her owner because he/she is threatened with death. This concern might be addressed by a requirement to incorporate explicit ethical considerations in extra-welfarist evaluation frameworks into the process of deliberation. It has been argued that ethical considerations are a productive heuristic to identify cooperation problems that can be overcome by the consenting institutions so that deliberation on a decision algorithm is also likely to benefit from such considerations from a purely constitutional economic perspective [80].

This study proposed an approach for a more considerate use of methods for preference elicitation, based on a constitution economic framework inspired by current approaches to order ethics. There remains a need for further theoretical research, e.g. ethical work to fully develop a deontological theory of healthcare prioritization or statistical work to develop a framework for equivalence testing in this normative context. Empirically, the feasibility of this approach and its suitability to arrive at the consensus that it envisages remain to be tested by further pilot studies implementing the approach.

## Conclusions

Methods for preference elicitation are widely used in healthcare. However, the normative bases of the most frequent uses have been criticized from an ethical perspective. This study proposed a theoretical framework that rests on a constitutional economic basis. It uses methods for preference elicitation with the aim of generating consensus about an evaluation framework in a two-step process: First, MCDA methods are used within a process of stakeholder deliberation to develop a tentative framework. Second, population-based methods for preference elicitation are used to assess whether a framework that can find consensus has indeed been obtained. This framework is compatible with welfarist economic theory, can incorporate extra-welfarist considerations about reasonable evaluation criteria, and may accommodate some deontological concerns because it rests on a contract ethical framework based on mutual respect and consent rather than on outcome maximization. Its application to establish indices of social value in health-related, ecological, or other fields of economics remains to be further explored.

## Data Availability

Not applicable for this literature-based study.

## Footnotes

1 This criterion requires that if individuals assign equal value to treating 10 people with cancer and 100 with knee damage, and to treating 10 people with knee damage and 100 with a headache, they should also assign equal value to treating 10 people with cancer and 1,000 people with a headache.
